# Let's talk about rings

**DOI:** 10.1186/1758-2946-5-S1-O8

**Published:** 2013-03-22

**Authors:** Matthias Rarey, Adrian Kolodzik, Sascha Urbaczek

**Affiliations:** 1 Center for Bioinformatics, University of Hamburg, Bundesstraße 43, 20146 Hamburg, Germany

## 

Defining the ring topology of a molecule belongs to the central and elementary problems of cheminformatics. Questions like 'How many rings does a molecule contain?' or 'In how many rings is a specific atom involved?' are based on such a definition. Obviously, the ring topology must be *unique*, i.e. it does not depend on atom order, *chemical meaningful*, and of *reasonable size*, at most polynomial in the number of atoms. For a long period, the smallest set of smallest rings (SSSR) was used in cheminformatics applications ignoring a very critical flaw, namely that it is not unique even for simple structures. Other definitions like the set of relevant cycles heal this flaw; however they are sometimes chemically not meaningful and can become exponential in size. Among all attempts made, none fulfils all three criteria at the same time [1].

Recently, we developed a ring definition named 'unique ring family' (URF) and a corresponding algorithm for calculating it in polynomial time [2]. URFs match the common chemical sense of a molecule's ring topology. The definition results in a unique ring description consisting of a number of ring prototypes which is at most quadratic in the number of atoms. In this talk, we will present the algorithm, benchmarks as well as several examples demonstrating the usefulness of URFs.
